# Cerebrospinal fluid pleocytosis level as a diagnostic predictor? A cross-sectional study

**DOI:** 10.1186/s12907-017-0053-0

**Published:** 2017-08-24

**Authors:** Anne Ahrens Østergaard, Thomas Vognbjerg Sydenham, Mads Nybo, Åse Bengård Andersen

**Affiliations:** 1 Department of Infectious Diseases, Odense University Hospital, Odense, Denmark; 2 Department of Clinical Microbiology, Odense University Hospital, Odense, Denmark; 3 Department of Clinical Biochemistry and Pharmacology, Odense University Hospital, Odense, Denmark; 4 University of Southern Denmark, Odense, Denmark; 5 Department of Infectious Diseases 8632, Copenhagen University Hospital Rigshospitalet, Blegdamsvej 9, DK 2100 Copenhagen OE, Denmark

**Keywords:** Cerebrospinal fluid, CSF, Pleocytosis, Lumbar puncture, Central nervous system infection, CNS infection, Seizures

## Abstract

**Background:**

Lumbar puncture with quantification of leukocytes and differential count of cellular subsets in the cerebrospinal fluid is a standard procedure in cases of suspected neuroinfectious conditions. However, a number of non-infectious causes may result in a low leukocyte number (0–1000 cells/ml). We wanted to assess the diagnostic diversity of unselected adult patients with pleocytosis in the cerebrospinal fluid.

**Methods:**

The study is based on data from cerebrospinal fluid (CSF) analyses of all adult patients (15 years or older) admitted to a large university hospital in Denmark during a two-year period (2008–2009). Data from the local patient administrative system supplied with data from patient charts were combined with laboratory data.

**Results:**

A total of 5390 cerebrospinal fluid samples from 3290 patients were included. Pleocytosis >5 leucocytes/μl was found in samples from 262 patients of which 106 (40.5%) were caused by infection of the central nervous system (CNS), 20 (7.6%) by infection outside CNS, 79 (30.2%) due to non-infectious neurological diseases, 23 (8.8%) by malignancy, and 34 (13.0%) caused by other conditions. Significantly higher mean CSF leukocytes was found in patients suffering from CNS infection (mean 1135 cells/μl, *p*-value <0.0001).

**Conclusions:**

CNS infection, non-infectious neurological disease, malignancy, and infection outside CNS can cause pleocytosis of the cerebrospinal fluid. Leukocyte counts above 100/μl is mainly caused by CNS infection, whereas the number of differential diagnoses is higher if the CSF leukocyte counts is below 50/μl. These conditions are most commonly caused by non-infectious neurological diseases including seizures.

## Background

Migration of leukocytes to the cerebrospinal fluid (CSF) is a cardinal symptom of an infectious condition affecting the meninges or the cerebral parenchyma. Bacterial and viral meningitis cannot reliably be differentiated clinically and requires lumbar puncture to analyse the CSF [1, 2]. Patients suffering from viral meningitis present CSF leukocyte concentrations varying from 10 to 1000/μl, but typically below 500 [3]. In bacterial meningitis CSF leukocytes vary from below 100 to more than 10,000 leukocytes/μl, often between 1000 and 5000/μl [4]. However, pleocytosis in the CSF may also occur in other medical conditions, e.g. neurological, rheumatic or malignant disease [5, 6]. Some patients with pleocytosis in the CSF never obtain a final diagnosis and in many settings the proportion of patients with “suspected CNS infection” is larger than that of patients with proven aetiology [1].

Apart from the issue of a large overlap in leukocyte concentrations caused by viral and bacterial infections, the relative distributions of the non-infectious diagnoses are not well described. One reason for this is the fact that these patients are dealt with by different clinical specialities.

## Methods

This study aimed at obtaining an overview of the relative contribution of the causes of cerebrospinal pleocytosis by a comprehensive method including all adult patients (regardless indication for lumbar puncture, requesting department, and symptomatology of the patient) admitted to a large university hospital in Denmark during a two-year period.

Pleocytosis is defined as increased cell count. In the following the term pleocytosis will be used to describe >5 leucocytes/μl in CSF. The study was performed at Odense University Hospital, a large regional hospital in Denmark with 1038 beds serving as referral hospital for 1.8 million inhabitants and holding all medical specialities including neurology, neurosurgery, rheumatology, oncology and haematology.

Data from CSF analyses performed at the Department of Clinical Biochemistry and Pharmacology from January 1st 2008 to December 31th 2009 (sample date, CSF leukocytes, CSF monocytes, CSF polymorphonuclear leukocytes, CSF protein, CSF glucose and plasma glucose) and data from the patient administration system of Funen County (FPAS; Fyns Patient Administrative System) (patient age, sex, hospital admission dates, discharge dates and discharge diagnoses) from 1994 to 2012 was retrieved. In addition, we obtained data from the electronic patient records on hospital admissions and CSF sample reports.

**Fig. 1:**
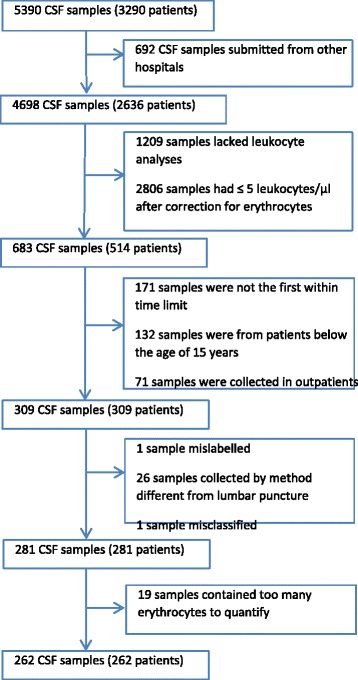
Flowchart of study inclusion

Only the initial CSF analysis requested with pleocytosis (>5 leukocytes/μl after correction for erythrocytes (1 leucocyte/1000 erythrocytes)) in hospitalized patients at age of 15 years or older were included. Exclusion criteria were: not the first CSF sample in the time period, wrong social security number, if the sample was not cerebrospinal fluid, if the erythrocytes were in layers or too numerous to quantify, if a sample was collected by a method different from lumbar puncture, or if the patient was transferred to another hospital with an uncertain diagnosis. If a sample was analysed more than once, the report given to the clinician was used (Fig. 1). Discharge diagnoses (ICD-10) were used to categorise the cause of pleocytosis. However, the cause of pleocytosis was adjusted in the following circumstances: 1) Discharge diagnosis was not verified para-clinically (magnetic resonance imaging (MRI)/computed tomography (CT), microbiological analyses, flow-cytometry, or autopsy) and the discharge summary mentioned a diagnosis in plain text not coded as a discharge diagnosis; the cause of pleocytosis was changed to this diagnosis. 2) a CNS infection was mentioned in the discharge summary but not included as a discharge diagnosis; Cause of pleocytosis was changed to *CNS infection*. 3) a discharge diagnosis was not verified nor was CNS infection but a secondary diagnosis was verified; the cause of pleocytosis was changed to this diagnosis. 4) Discharge diagnosis was “Observation for other suspected diseases and conditions” or “Observation for suspected nervous system disorder” and the suspected disorder was CNS infection; the cause of pleocytosis was changed to *CNS infection*, 5) the patient received full treatment for a CNS infection but this was not included in discharge summary; the cause of pleocytosis was changed to *CNS infection.* 6) the discharge diagnosis did not coincide with the patient chart or discharge summary; the cause of pleocytosis was changed to the cause mentioned in the chart. 7) a clear diagnosis was not made at time of discharge but was verified within 3 months after discharge date; this diagnosis was used as cause of pleocytosis.

The category *CNS infection* was further divided into 2 groups: *verified* and *probable*. A discharge diagnosis was considered *verified* if the diagnosis was verified by MRI, CT, microbiological analyses, or autopsy.

Charlson index score [7] was used to describe patients comorbidity. Charlson score estimating risk of death from comorbid disease in longitudinal studies by scoring each comorbid disease a score of 1, 2, 3, or 6.22 comorbidity conditions are included. Calculation was based on registered diagnoses in the patient administration system from years 1994 to 2009.

### Statistical analysis

Data processing was performed in STATA version 13.1–14.0. Wilcoxon/Mann-Whitney (Mann-Whitney U/Wilcoxon rank sum) test was performed for data not normally distributed (sex, Charlson score, CSF leukocytes, CSF protein, CSF monocyte proportion, and CSF polymorph nuclear leukocyte proportion). Student’s t-test was used if data was normally distributed (age). All *p*-values were comparisons between the marked category and the other categories. Only significant *p*-values (≤0.05) were included.

## Results

Out of 5390 unselected cerebrospinal fluid samples, 262 met the inclusion criteria (Fig. 1). The neurological department (56.5%), department of emergency admission (14.9%), and the department of intensive care (7.3%) were the main contributors to CSF analyses.

Patients were divided into diagnosis categories of *CNS infection*, *Infection outside CNS*, *Non-infectious neurological diseases*, *Malignancy*, *Other*, and subgroups (Appendix A). *CNS infection* amounted to 40.5% of the causes of pleocytosis. *Infection outside CNS* to 7.6%, *Non-infectious neurological diseases* to 30.2%, *Malignancy* 8.8%, and *Other* 13.0%.

There was no significant difference in distribution of sex in any of the categories (Table 1). Significantly lower mean age was found in the *Non-infectious neurological diseases* category (mean age 46.1, *p*-value 0.0088). Mean Charlson score in the 262 patients was 0.9, range 0–11. Mean Charlson score was significantly lower in the *Non-infectious neurological diseases* category (mean 0.6, *p*-value 0.0125), while it was significantly higher in the *Malignancy* (mean 2.7, *p*-value <0.001) than in all other categories. In general, there was a tendency of high mean Charlson score in the categories with high mean age.

**Table 1:** Summery of baseline findings by diagnosis category

	Diagnosis category											
	CNS infection (*n* = 106)		Infection outside CNS (*n* = 20)		Non-infectious neurological diseases (*n* = 79)		Malignancy, all foci (*n* = 23)		Other (*n* = 34)		Total (*n* = 262)	
Sex^a^	n (%)		n (%)		n (%)		n (%)		n (%)		n (%)	
Male	49 (46.2)		11 (55.0)		43 (54.4)		14 (60.9)		16 (47.1)		133 (50.8)	
Female	57 (53.8)		9 (45.0)		36 (45.6)		9 (39.1)		18 (52.9)		129 (49.2)	
Age^b^	n (%)		n (%)		n (%)		n (%)		n (%)		n (%)	
< 36 yrs	26 (24.5)		5 (25.0)		24 (30.6)		2 (8.7)		7 (20.6)		64 (24.4)	
36-51 yrs	31 (29.3)				27 (34.2)		6 (26.1)*		7 (20.6)		71 (27.1)	
52-64 yrs	20 (18.9)		4 (20.0)		14 (17.7)		9 (39.1)		13 (38.2)		60 (22.9)	
≥ 65 yrs	29 (27.4)		11 (55.0)		14 (17.7)		6 (26.1)		7 (20.6)		67 (25.6)	
Mean		50.4		58.5		46.1*		59.1		52.0		50.7
Range		15–88		17–91		20–89		28–81		17–95		15–95
Charlson score^a^	n (%)		n (%)		n (%)		n (%)		n (%)		n (%)	
0	66 (62.3)		9 (45.0)		52 (65.8)		1 (4.4)		19 (55.9)		147 (56.1)	
1	25 (23.6)		3 (15.0)		18 (22.8)		1 (4.4)		9 (26.5)		56 (21.4)	
2	7 (6.6)		5 (25.0)		5 (6.3)		14 (60.9)		6 (17.7)		37 (14.1)	
3	2 (1.9)		2 (10.0)		2 (2.5)		3 (13.0)				9 (3.4)	
≥4	6 (5.7)		1 (5.0)		2 (2.5)		4 (17.4)				13 (5.0)	
Mean		0.7		1.2		0.6		2.7**		0.6		0.9
Range		0–6		0–4		0–7		0–11		0–2		0–11

^a^Wilcoxon-Mann-Whitney test

^b^ *p*-values calculated by Student’s t-test

*p-value <0.01

**p-value <0.001

In 141 patients mean CSF/plasma glucose ratio was 0.6 (normal). Significantly lower CSF/plasma glucose ratio was found in the decreased interval (<0.46) for *CNS infection* (mean 0.2, *p* 0.0085). Significant higher CSF/plasma glucose ratio was found in the decreased interval (<0.46) for *Non-infectious neurological diseases* (mean 0.4, *p* 0.0276).

The mean concentration of CSF leukocytes was 494/μl (Table 2). Significantly higher concentrations of mean CSF leukocytes were found in patients with *CNS infection* (mean 1135, *p*-value <0.001). In the category *CNS infection* no distinction between viral and bacterial neuroinfection was made. When only verified diagnoses were included, a higher concentration of CSF leukocytes were found in the category *Meningitis, acute bacterial* (*p*-value = 0.0002) (Table 5) compared to the others in *CNS infection*.

**Table 2:** Summary of CSF findings by diagnosis category

	Diagnosis category											
	CNS infection (*n* = 106)		Infection outside CNS (*n* = 20)		Non-infectious neurological diseases (*n* = 79)		Malignancy, all foci (*n* = 23)		Other (*n* = 34)		Total (*n* = 262)	
CSF leukocytes/μl^a^	n (%)	[mean]	n (%)	[mean]	n (%)	[mean]	n (%)	[mean]	n (%)	[mean]	n (%)	[mean]
6–10	11 (10.4)	8.1	10 (50.0)	7.3	32 (40.5)	7.3	8 (34.8)	7.4	14 (41.2)	7.2	75 (28.6)	7.4
>10–50	23 (21.7)	29.3	9 (45.0)	22.9	34 (43.0)	20.2	11 (47.8)	21.9	14 (41.2)	20.1	91 (34.7)	23.0
>50–100	8 (7.6)	84.0			3 (3.8)	66	2 (8.7)	62.5	5 (14.7)	69.8	18 (6.9)	74.7
>100–200	17 (16.0)	135.0			6 (7.6)	138			1 (2.9)	159	24 (9.2)	136.8
>200–400	13 (12.3)	266.4	1 (5.0)	221	2 (2.5)	247.5	1 (4.4)	347			17 (6.5)	266.2
>400–600	12 (11.3)	524.6			1 (1.3)	537					13 (5.0)	525.5
>600–800	2 (1.9)	744									2 (0.8)	744
>800–1000	3 (2.8)	896									3 (1.2)	896
>1000	17 (16.0)	6041.2			1 (1.3)	2015	1 (4.4)	1980			19 (7.3)	5615.5
Total	106 (100.0)	1135.5**	20 (100.0)	25.0*	79 (100.0)	63.2**	23 (100.0)	119.7	34 (100.0)	26.2*	262 (100.0)	494.3
CSF protein g/l^a^	n (%)	[mean]	n (%)	[mean]	n (%)	[mean]	n (%)	[mean]	n (%)	[mean]	n (%)	[mean]
<0.2					1 (1.3)	0.0			1 (2.9)	0.0	2 (0.8)	0.0
0.2–0.4	18 (17.0)	0.4	12 (60.0)	0.3	34 (43.0)	0.3	8 (34.8)	0.3	11 (32.4)	0.4	83 (31.7)	0.3
0.41–1.11	50 (47.2)	0.7	8 (40.0)	0.8	36 (45.6)	0.7	10 (43.5)	0.6	18 (52.9)	0.6	122 (46.6)	0.7
>1.11	38 (35.9)	2.8			8 (10.1)	2.5	5 (21.7)	2.9	4 (11.8)	2.6	55 (21.0)	2.8
Total	106 (100.0)	1.4**	20 (100.0)	0.5	79 (100.0)	0.7**	23 (100.0)	1.0	34 (100.0)	0.7	262 (100.0)	1.0

^a^Wilcoxon-Mann-Whitney test

^b^ *p*-values by Student’s t-test

*p-value <0.01

**p-value <0.001

**Fig. 2:**
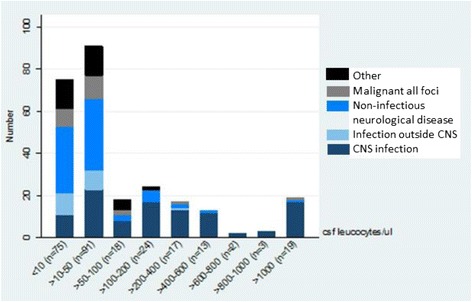
Distribution of diagnose category per cell count. CNS infection is the only category present in all intervals. The category of Other occurs mainly in patients with CSF leukocytes below 100/μl. Infection outside CNS occurs primarily when CSF leukocytes were below 50/μl

The proportion of patients in the *CNS infection* category increased with increasing CSF leukocyte concentration, and at leukocyte counts above 100/μl *CNS infection* was the most frequent cause of pleocytosis (Fig. 2). Eighty seven point three percent of the patients with more than 200 leukocytes were diagnosed with *CNS infection.*

**Fig. 3:**
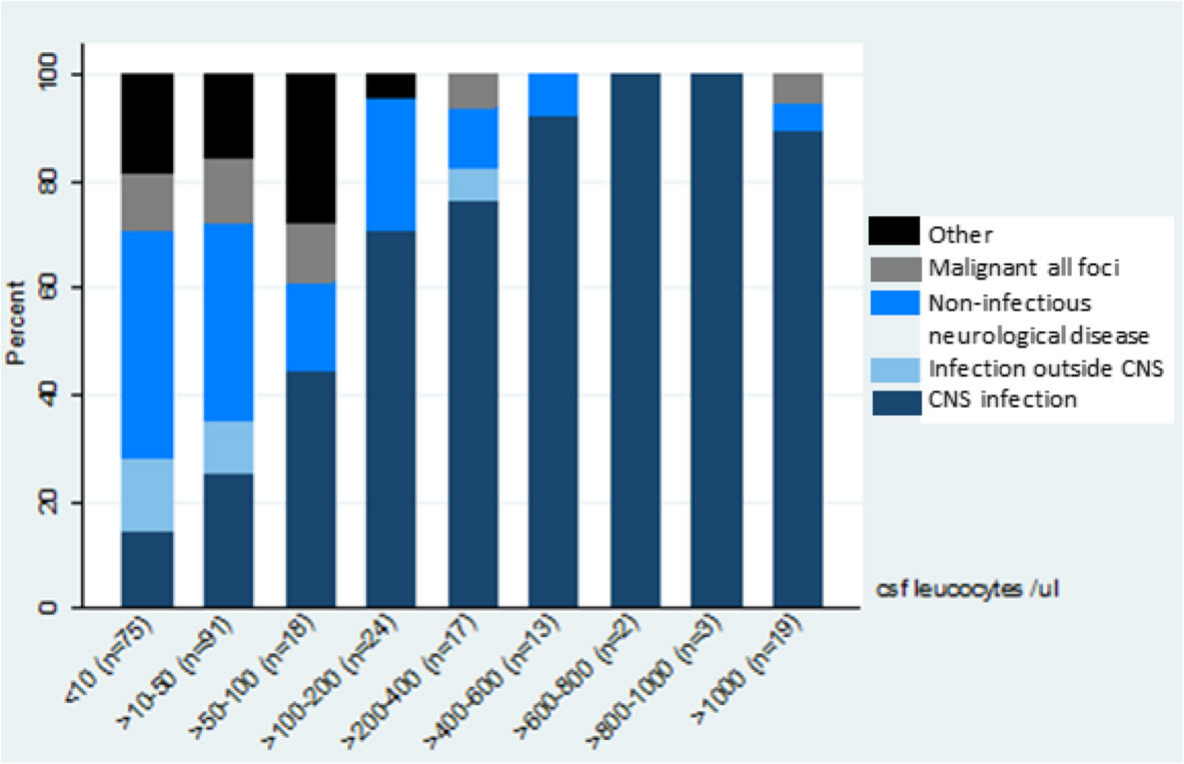
Distribution of diagnose category as a percentage per cell count. The proportion of patients in the *CNS infection* category increased with increasing CSF leukocyte concentration, and at leukocyte counts above 100/μl *CNS infection* was the most frequent cause of pleocytosis

In CSF samples with leukocytes below 50/μl especially non-infectious neurological diagnoses should be considered as differential diagnoses, since the proportion of *CNS infection* increased with increasing CSF leukocytes (Fig. 3). The category of *Other* seemed to occur mainly in patients with CSF leukocytes below 100/μl. *Infection outside CNS* occurred primarily when CSF leukocytes were below 50/μl. *CNS infection* was the only category present in all intervals.

The mean CSF protein concentration in all patients was 1.0 g/l (Table 2). CSF protein was normal (0.2–0.4 g/l) or decreased in 32.4% of all patients. All patients with *CNS infection* had a significantly higher level of protein in CSF (mean 1.4) (*p*-value <0.0001). CNS protein is known to be increased in meningitis, but also to be one of the least specific parameters in CSF [4].

Of the 106 patients with *CNS infection* 59 (55.7%) were paraclinically confirmed. For the categories of *malignancy* 20 (87.0%), *other* 15 (44.1%), *non-infectious neurologically disease* 37 (46.8%), and *other infection* 10 (50.0%) were paraclinically confirmed. Table 3 shows that more diagnoses in the CNS infection category were paraclinically verified as cell count increases. Mean protein level was higher in the verified CNS infections than in probable CNS infection. CSF/plasma glucose ratios shows a tendency of being lower in total in the verified category and are in both groups the lowest when cell count is high.

**Table 3:** CSF leukocyte count, mean protein and mean CSF/plasma glucose ratio in verified and probable CNS infection

CSF leukocytes /μl a	CNS infection, Verified			CNS infection, Probable			Total		
	n (% horizontal)	Protein g/l Mean (range)	Glucose ratio mean (range)	n (% horizontal)	Protein g/l mean (range)	Glucose ratio mean (range)	n (% vertical)	Protein g/l mean (range)	Glucose ratio mean (range)
6–10	4 (36.4)	0.6 (0.4–0.9)	-	7 (63.6)	0.7 (0.4–0.9)	0.7 (0.5–0.9)	11 (10.4)	0,6 (0.4–0.9)	0.7 (0.5–0.9)
>10–50	9 (39.1)	0.9 (0.3–2.2)	0,5 (0.4–0.7)	14 (60.9)	0.6 (0.3–2.2)	0.6 (0.3–1.0)	23 (21.7)	0.7 (0.2–2.2)	0.6 (0.3–1.0)
>50–100	5 (62.5)	1.0 (0.2–1.6)	0.6 (0.5–0.7)	3 (37.5)	0.6 (0.4–0.7)	0.4 (0.4–0.5)	8 (7.6)	0,8 (0.2–1.6)	0.5 (0.4–0.7)
>100–200	7 (41.2)	0.8 (0.3–1.3)	0.7 (0.5–0.7)	10 (58.8)	0.8 (0.4–1.7)	0.6 (0.6–0.8)	17 (16.0)	0.8 (0.3–1.7)	0.7 (0.6–1.0)
>200–400	8 (61.5)	1.3 (0.4–2.4)	0.5 (0.4–0.6)	5 (38.5)	0.9 (0.5–1.9)	0.6 (0.5–0.8)	13 (12.3)	1.1 (0.4–2.4)	0.6 (0.4–0.8)
>400–600	8 (66.7)	2.3 (0.7–6.8)	0.4 (0.0–0.6)	4 (33.3)	1.3 (0.6–2.8)	0.6 (0.4–0.9)	12 (11.3)	2.0 (0.6–6.8)	0.5 (0.0–0.9)
>600–800	2 (100.0)	1.4 (0.8–2)	0.5 (0.3–0.6)				2 (1.9)	1.4 (0.8–2.0)	0.5 (0.3–0.6)
>800–1000	2 (66.7)	1.2 (1.2–1.2)	0.7 (0.7–0.7)	1 (33.3)	1.4	0.4	3 (2.8)	1.3 (1.2–1.4)	0.5 (0.4–0.7)
>1000	14 (82.4)	4 (0.8–6.8)	0.1 (0.1–0.1)	3 (17.6)	4.4 (1.5–9.0)	0.2 (0.1–0.1)	17 (16.0)	3.6 (0.8–6.8)	0.2 (0.0–0.4)
Total	59 (55.7)	1.8 (0.2–6.8)	0.4 (0.0–1.0)	47 (44.3)	1.0 (0.2–9.0)	0.6 (0.1–1.0)	106 (100.0)	1.4 (0.2–9)	0.5 (0.0–1.0)

- missing data: CSV/plasma glucose ratio only available in 59 of 106 patients

For six patients, seizures, epilepsy or status epilepticus was the cause of pleocytosis. Two patients had <10 leukocytes/μl and four patiens had 10–50 leukocytes/μl (Table 4). Pleocytosis has previosly been found in patientes after seizures [8]. Higher concentrations of CSF leukocytes were found in *Other neurological diseases*. One patient, who suffered from obstructive hydrocephalus, had >1000 leukocytes/μl in CSF (categorised as *Other neurological diseases*) but did not suffer from neuroinfection. No distinction between viral and bacterial meningitis was made. No patients had tuberculosis or fungal CNS infection as shown in the Appendix.

**Table 4:** CSF leukocyte count in non-infectious neurological diseases subgroups

Non-infectious neurological diseases subgroup	CSF leukocytes/μl							
	≤10	>10–50	>50–100	>100–200	>200–400	>400–600	>600	Total
	n (%)	n (%)	n (%)	n (%)	n (%)	n (%)	n (%)	n (%)
Encephalitis/myelitis, non-infectious		8 (66.7)		2 (16.7)	1 (8.3)	1 (8.3)		12 (100.0)
Seizures/epilepsy/status epilepticus	2 (33.3)	4 (66.7)						6 (100.0)
Ischemia/infarction/stroke	4 (36.3)	4 (36.6)	1 (9.1)	2 (18.2)				11 (100.0)
Intracranial haemorrhage		1 (50.0)		1 (50.0)				2 (100.0)
Multiple sclerosis	8 (53.3)	7 (46.7)						15 (100.0)
Demyelinating disease/polyneuropathy	2 (40.0)	3 (60.0)						5 (100.0)
Paralysis/palsy of cranial nerve	2 (66.7)		1 (33.3)					3 (100.0)
Headache/migraine	3 (60.0)	2 (40.0)						5 (100.0)
Other neurological	11 (55.0)	5 (25.0)	1 (5.0)	1 (5.0)	1 (5.0)		1 (5.0)	20 (100.0)
Total	32 (40.5)	34 (43.0)	3 (3.8)	6 (7.6)	2 (2.5)	1 (1.3)	1 (1.3)	79 (100.0)

## Discussion

The discharge diagnoses were retrospectively adjusted for 36 (13.7%) patients following discharge summary and patient chart review. We found it important to manually review the charts as also suggested by others in retrospective studies to secure that all relevant diagnoses were included [9, 10].

A wide span of diagnoses were included in the *Others* category including neurosarcoidosis and rheumatic diseases, which are known causes of pleocytosis [6, 11–13]. In the category *Other,* the mean CSF leukocyte concentration was 26/μl (SD 32.6). The patients in this category did not differ from the other patients in age, sex, or Charlson score. It could be speculated that some of these patients suffered from a benign viral infection either not detected by available diagnostic setups or not tested for.

In the *Non-infectious neurological diseases* category five patients suffered from *Migraine* or *Headache* and three patients from *Paralysis/paresis of facial nerve*. These eight patients could have suffered from a mild viral CNS infection or Lyme’s disease, since viral CNS infection can present with similar symptoms [14, 15]. However, variation from the normal levels cannot be ruled out.

Three patients in the *Malignancy* category suffered from lung or oropharyngeal cancer. Previously, it has been found that patients can develop chemical meningitis (sterile and inflammatory) due to concurrent systemic and local chemotherapy [16]. This could explain pleocytosis in these patients. One patient in the *Malignancy* category was found with 1980 cells in CSF and suffered from agranulocytosis secondary to cancer chemotherapy but was not found to suffer from a CNS infection though the high cell count would suggest otherwise. Neither was the patient found to suffer from infection else where and was therefore categorized as *Malignancy*. This explains the high mean cell count in the subgroup *Cancer, foci elsewhere* (Table 5).

### Limitations

The study was performed retrospectively, which means that no actions of the treating staff or patients could influence the results. A limitation of the study is the fact that data only was available from registers and patient charts, which might have led to incorrect categorization of some of the patients. However, all patients charts have been reviewed and categorized as part of this study as described in the methods section. Not all diagnoses were paraclinically confirmed. This could be due to administration of antimicrobial therapy prior to lumbar puncture or due to insufficient sensitivity of the available methods.

## Conclusions

This study correlates CSF findings to final diagnosis. CNS infection, non-infectious neurological disease, malignancy, and infection outside CNS can cause pleocytosis of the cerebrospinal fluid. Leukocyte counts above 100/μl are mainly caused by CNS infection, whereas the number of differential diagnoses is higher when CSF leukocytes levels are below 50/μl. These conditions are most commonly caused by non-infectious neurological diseases including seizures.

## Appendix

**Table 5:** Summary of subgroups in the diagnosis categories

	Number	Percent	Mean CSF leukocytes/μl
CNS infection	106	40.5	1135.5
Meningitis. viral/unknown agent	38	35.9	617.0
Meningitis. acute bacterial	21	19.8	3591.3
Meningitis. borrelia/syphilis	25	23.6	150.6
Encephalitis. infectious	11	10.4	295.3
CNS abscess	3	2.8	4654.3
Other CNS infection (Including: Herpes zoster with other complication in CNS, Hydrocephalus caused by infectious or parasitic disease classified elsewhere, Meningoencephalitis due to toxoplasmosis, Polyneuropathy caused by infectious or parasitic disease classified elsewhere, Viral infection of CNS unspecified)	8	7.6	66.0
Infection outside CNS	20	7.6	25.0
Endocarditis	2	9.5	113.5
Sepsis	10	47.6	18.8
Other infection with foci outside CNS (including: Acute pharyngitis, Acute sinusitis, Analabsces, Other pneumonia due to other unspecified microorganism, Oral candidiasis, HIV with other infectious and parasitic disease, Erysiphelas, unspecified, Viralinfection, unspecified, Pilonidal cyst with abscess)	8	40.0	10.6
Non-infectious neurological diseases	79	30.2	63.2
Encephalitis/myelitis. Non-infectious	12	15.2	108.3
Seizures/epilepsy/status epilepticus	6	7.6	15.8
Ischemia/infarction/stroke	11	13.9	38.9
Intracranial haemorrhage	2	2.5	85.0
Multiple sclerosis	15	19.0	15.0
Demyelinating disease/polyneuropathy	5	6.3	10.8
Paralysis/palsy of cranial nerve	3	3.8	29.7
Headache/migraine	5	6.3	10.2
Other neurological (including: Acute transverse myelitis in demyelinating disease of central nervous system, Other extrapyramidal and movement disorders, Ventriculitis of the brain, unspecified, Sequelae of inflammatory diseases of central nervous system), Obstructive hydrocephalus, Spinal stenosis, Herniation of lumbar disc with radiculopathy, Disorder of central nervous system, unspecified, Symptom of central nervous system unspecified)	20	25.3	129.1
Malignancy. all foci	23	8.8	119.7
Lymphoma	9	39.13	20.7
Leukaemia	3	13.04	16.3
CNS cancer	5	21.74	30.6
Cancer. foci elsewhere (Including: Malignant neoplasm of unspecified part of unspecified bronchus or lung, Malignant neoplasm of oropharynx, unspecified, Agranulocytosis secondary to cancer chemotherapy)	4	17.39	500.5
Carcinomatosis	2	8.7	181.0
Other	34	13.0	26.2
Sarcoidosis	4	11.4	54.5
Rheumatologic	2	5.7	89.0
Cardiologic. non-infectious	2	5.7	8.0
Observation for suspected/other or not specified findings	8	22.9	25.9
Others (Including: Acute respiratory failure, Fever, unspecified, Hypokalemia, Complications following infusion, transfusion and therapeutic injection, Care involving use of rehabilitation procedure, unspecified, Unspecified multiple injuries, Hypopituitarism, Benign paroxysmal vertigo)	18	52.9	15.1

## Abbreviations

CNS: Central nervous system
CSF: Cerebrospinal fluid
CT: Computed tomography
MRI: Magnetic resonance imaging
PCR: Polymerace chain reaction
